# Supplementary material for: Effects of the COVID-19 Pandemic on Legionella Water Management Program Performance across a United States Lodging Organization
Source: Int J Environ Res Public Health. 2023 Oct 5;20(19):6885. doi: 10.3390/ijerph20196885 (PMC10572137; doi:10.3390/ijerph20196885)
Supplement: Supplementary file 1 [file ijerph-20-06885-s001.zip › ijerph-2541450-supplementary.pdf]

Article

Effects of the COVID-19 Pandemic on *Legionella* Water Management Program Performance across a United States Lodging Organization

Jasen M. Kunz [1,*], Elizabeth Hannapel [2], Patrick Vander Kelen [3], Janie Hils [3,4], Edward Rickamer Hoover [3] and Chris Edens [2]

Supplementary Materials

Table S1. Environmental Samples Tested for *Legionella* During the COVID-19 Pandemic vs. Before by Period of Detection Among Facilities with any *Legionella* Detection

	Before Pandemic		During Pandemic		Total	
Period of *Legionella* Detection at Facilities	Negative Samples	Positive Samples	Negative Samples	Positive Samples	Negative Samples	Positive Samples
Only before the pandemic (n=55)	916 (86%)	148 (14%)	380 (100%)	0 (0%)	1,296 (90%)	148 (10%)
Both before and during the pandemic (n=94)	1,984 (78%)	566 (22%)	939 (63%)	544 (37%)	2,923 (72%)	1,110 (28%)
Only during the pandemic (n=81)	1,247 (100%)	0 (0%)	719 (67%)	350 (33%)	1,966 (85%)	350 (15%)
Total (n=230)	4,147 (85%)	714 (15%)	2,038 (70%)	894 (30%)	6,185 (79%)	1,608 (21%)

Table S2. Environmental Samples Tested for *Legionella* by *Legionella* Type Result, Water System, and COVID-19 Pandemic Time Period

		SG1 OR Non-SG1		SG1		Non-SG1	
Water System	Time Period	Negative Samples	Positive Samples	Negative Samples	Positive Samples	Negative Samples	Positive Samples
Hot and Cold Water[1]	Before Pandemic	10594 (94%)	714 (6%)	11126 (98%)	182 (2%)	10757 (95%)	551 (5%)
	During Pandemic	5527 (86%)	894 (14%)	6192 (96%)	229 (4%)	5720 (89%)	701 (11%)
Hot Water	Before Pandemic	6342 (92%)	583 (8%)	6778 (98%)	147 (2%)	6473 (93%)	452 (7%)
	During Pandemic	3080 (84%)	593 (16%)	3520 (96%)	153 (4%)	3208 (87%)	465 (13%)
Cold Water	Before Pandemic	3360 (97%)	91 (3%)	3429 (99%)	22 (1%)	3380 (98%)	71 (2%)
	During Pandemic	1960 (89%)	245 (11%)	2151 (98%)	54 (2%)	2004 (91%)	201 (9%)

[1] Data contains all samples, including those of unknown water system (i.e., samples not labelled as hot or cold).

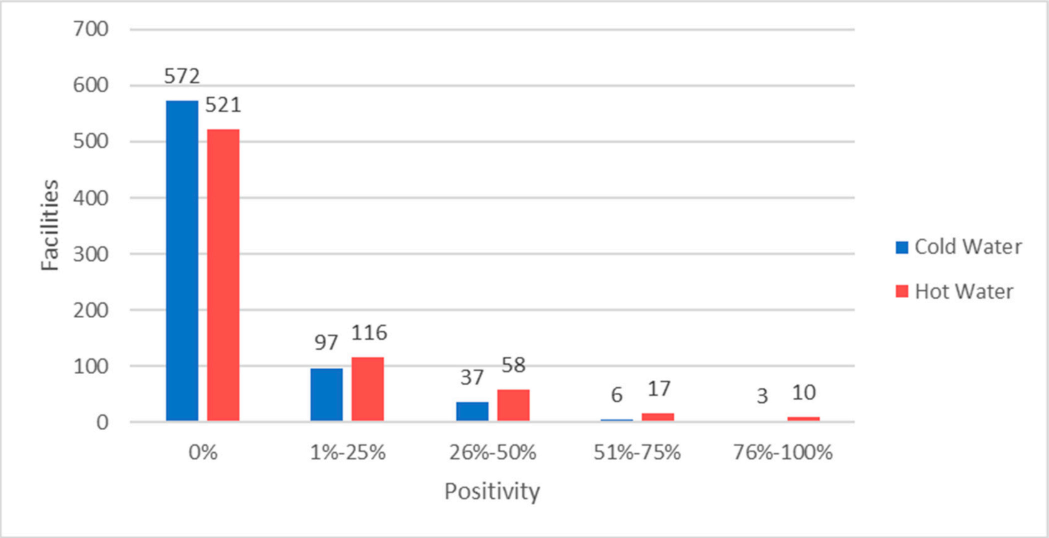

Figure S1. Number of Facilities by *Legionella* Percent Positivity in Cold and Hot Water

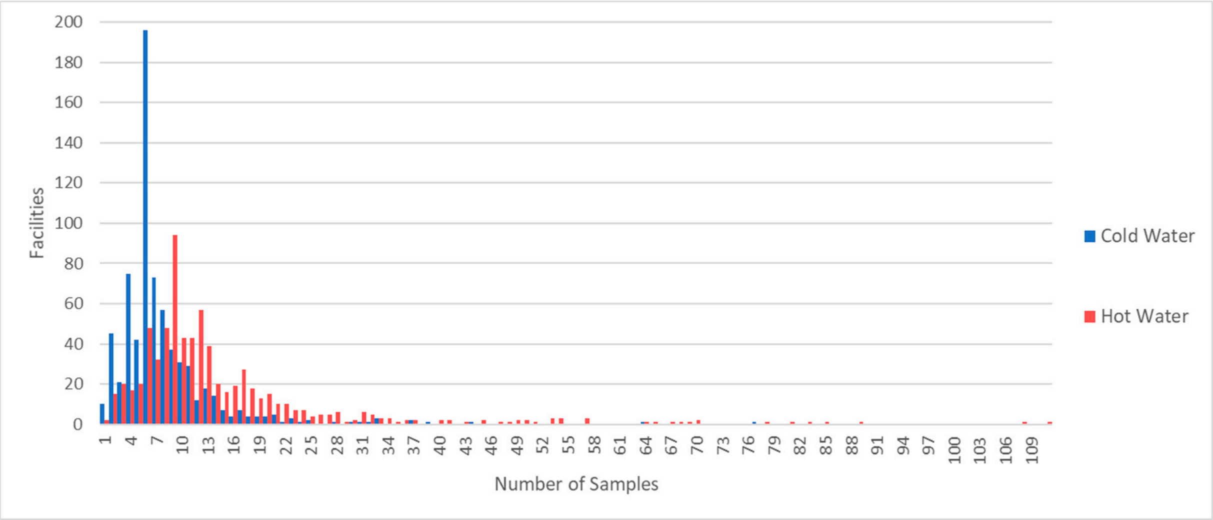

Figure S2. Number of Facilities by Number of Environmental Samples Tested in Cold and Hot Water
